# Prevalence, predictors, barriers and facilitators of self-monitoring of blood glucose in patients with type 2 diabetes: A multicentre cross-sectional study

**DOI:** 10.51866/oa.1058

**Published:** 2026-05-18

**Authors:** Mervyn Eng Han Ong, Xian Goo Yong, Kasturi Mahalinggam, Yee Ling Tan, Noor Asyila Ismail, Marni Raihana Yusof, Priyadarshini Ramachandran, Hooi Min Lim

**Affiliations:** 1 Klinik Kesihatan Beranang, Jalan Semenyih-Seremban, Beranang, Selangor Malaysia.; 2 Department of Primary Care Medicine, Faculty of Medicine, Universiti Malaya, Kuala Lumpur, Malaysia.; 3 Klinik Kesihatan Putrajaya Presint 9 1, Jalan P9e, Presint 9, Putrajaya Wilayah Persekutuan Putrajaya, Malaysia.; 4 Klinik Kesihatan Bandar Tun Hussein Onn Jalan Suadamai, Bandar Tun Hussein Onn, Cheras, Selangor, Malaysia.; 5 Klinik Kesihatan Kajang Jalan Semenyih, Kajang, Selangor, Malaysia.; 6 Klinik Segara Mercu UEM, Jalan Stesen Sentral 5, Kuala Lumpur, Malaysia.

**Keywords:** Blood glucose self-monitoring, Type 2 diabetes mellitus, Insulin, Diabetes complications

## Abstract

**Introduction:**

Self-monitoring of blood glucose (SMBG) is vital for diabetes self-care, but its uptake in Malaysia remains low. This study aimed to determine the prevalence, predictors, barriers and facilitators of SMBG among patients with type 2 diabetes in Malaysian primary care settings.

**Methods:**

A cross-sectional study was conducted from May to September 2024 across five urban, suburban and rural primary care clinics. Participants completed a validated questionnaire on sociodemographic characteristics, clinical characteristics and SMBG practices. Multivariable logistic regression was conducted to identify the predictors of SMBG.

**Results:**

Among the 396 participants (mean age=53.6±10.9 years), the prevalence of SMBG was 59.3%. The participants aged 50–59 years (adjusted odds ratio [AOR]=2.80, 95% confidence interval [CI] = 1.03-7.62, P=0.045) and 60-69 years (AOR=3.43, 95% CI=1.25-9.38, P=0.017) were more likely to perform SMBG than those aged ≥70 years. Insulin use was strongly associated with SMBG (A0R=7.02, 95% CI=2.44-20.19, P<0.001), whereas the presence of diabetes complications was negatively associated with SMBG (A0R=0.57, 95% CI=0.33-0.98, P=0.043). The major barriers were the cost of test strips and lancets (59.6%) and frustration with high glucose readings (82.8%), while the facilitators included personal motivation (68.9%), family support (79.1%) and belief in the importance of SMBG (86.9%).

**Conclusion:**

The prevalence of SMBG is moderate but suboptimal. Enhancing structured diabetes education and addressing financial barriers are essential to improve SMBG practice and glycaemic control.

## Introduction

Diabetes mellitus (DM) is a serious public health issue worldwide with its increasing prevalence in both developing and developed countries. As per the International Diabetes Federation, diabetes affected 9.3% (463 million) of adults globally in 2019,^1^ and this number is projected to rise to 10.2% (578 million) by 2030 and 10.9% (700 million) by 2045 if effective preventive measures are not implemented.^1^ Malaysia bears one of the highest burdens of diabetes in the Western Pacific region, with the prevalence rising from 11.2% in 2011 to 18.3% in 2019, reflecting a 68.3% increment.^2^ A national survey conducted in 2023 showed that 1.9 million Malaysian adults aged 18 years and above had diabetes, and this figure was expected to reach 7 million by 2025, causing a serious health and socioeconomic burden with a projected prevalence of 31.3%.^3,4^ Poor glycaemic control remains common in Malaysia, as evidenced by the National Diabetes Registry 2023, which reported that only 34% of patients with type 2 diabetes (T2D) in clinics under the Ministry of Health achieved HbA1c levels of ≤6.5%.^3^ Large registry-based studies demonstrated that 72.7% of patients in Kedah^5^ and 68% of patients in Johor^6^ had uncontrolled T2D (HbA1c levels of ≥6.5%), indicating suboptimal diabetes management in Malaysia despite the availability of treatment and monitoring resources.

Self-monitoring of blood glucose (SMBG) plays a vital role in diabetes self-management, explicitly for patients treated with insulin, and is encouraged for patients on oral glucose-lowering drugs. It provides real-time feedback, supports treatment adherence and allows timely therapeutic adjustments to improve glycaemic control.^7^ Despite evidence showing that regular SMBG has been associated with significant HbA1c reduction^8^ and clinical guideline recommendations supporting its use,^9,10^ its uptake in Malaysia remains low; only 15.3% of patients in government clinics have reported practising SMBG.^11^ Inadequate adoption of regular SMBG contributes to suboptimal glycaemic control, predisposing patients to diabetes-related complications, reduced quality of life and increased healthcare expenditure in Malaysia.^3,12^

Although some studies have evaluated SMBG interventions in Malaysia and qualitative work has identified barriers among insulin users,^12,13^ data on real-world SMBG practices across diverse Malaysian primary care settings that cover private, urban and rural clinics remain scarce. Understanding the determinants of SMBG practice across diverse primary care settings is crucial to optimise diabetes self-management and guide targeted interventions. This study aimed to determine the prevalence and predictors of SMBG practice among patients with T2D in Malaysian primary care settings and to explore the perceived barriers and facilitators affecting SMBG practice.

## Methods

We conducted a cross-sectional questionnaire survey from May to September 2024 in five primary care clinics: Klinik Kesihatan (KK) Beranang, KK Bandar Tun Hussein Onn, KK Kajang, KK Putrajaya Presint 9 and Klinik Segara, KL Sentral. Ethical approval was granted by the Medical Research Ethics Committee, National Medical Research Register (NMRR ID-24-01346-UMG).

Three government clinics located in Hulu Langat, Selangor, one clinic in Putrajaya and one private general practice clinic in Kuala Lumpur were selected. These clinics represented rural (KK Beranang), suburban (KK Bandar Tun Hussein Onn) and urban (KK Kajang, KK Putrajaya Presint 9 and Klinik Segara) populations to capture a representative spectrum of the Malaysian primary care landscape. This selection strategy aimed to enhance the generalisability of the findings across different socioeconomic and geographic characteristics.

### Study participants

Patients aged over 18 years with T2D who attended the selected clinics during the study period were recruited. We excluded patients with type 1 DM, pregnancy, cognitive impairment, an inability to read Malay or English or critical illness hindering participation. The sample size was calculated using the Kish formula based on a prevalence of SMBG practice of 66%.^7^ With a confidence interval (CI) of 95%, a sample size of 396 patients was needed for this study.

### Study instrument

Data were collected using a self-administered questionnaire. This instrument was adapted from a validated tool from the study by Mastura et al.^11^ with permission, supplemented with 13 items on barriers and facilitators from the study by Ong et al.^13^ The final questionnaire was translated into Malay language according to the ISPOR guidelines.^14^ Although a new Cronbach’s alpha was not calculated for this cohort, the instrument underwent face validation through a pilot study with 30 participants to ensure linguistic clarity and contextual appropriateness.

The questionnaire consisted of four sections. The first section captured sociodemographic characteristics (age, sex, ethnicity, marital status, educational level, average monthly household income, smoking status and physical activity). The average monthly household income was categorised according to the national classification in Malaysia: B40 (<RM 5250), M40 (RM 5250-11,819) and T20 (>RM 11,819).

The second section addressed clinical characteristics, including duration of diabetes, current treatment regimen, diabetes-related complications (cardiac, renal, foot, ocular or neurological), history of diabetes-related hospitalisation and diabetes education. Diabetes education was defined as the successful completion of a formal education session conducted by a doctor or a certified diabetes nurse. Diabetes treatment regimens comprised lifestyle only, tablets only and the use of insulin.

The third section assessed SMBG practices: practice of SMBG, recording and treatment adjustments based on SMBG results. The practice of SMBG was defined as the self-reported performance of blood glucose monitoring at any frequency and any point of time.

The final section of the questionnaire consisted of 13 items assessing barriers and facilitators to SMBG. Each item was rated on a 5-point Likert scale, ranging from 1 *(strongly disagree)* to 5 *(strongly agree).* Higher scores indicated stronger agreement with the barrier or facilitator described.

### Data collection

Participants were recruited through convenience sampling at the triage counters of the selected clinics. Recruitment was conducted during clinic operating hours on weekdays at the researchers’ convenience. The researchers approached consecutive patients waiting for their consultations. Eligible patients were provided with a patient information sheet and given the opportunity to ask questions. Written informed consent was obtained prior to enrolment, and consenting patients completed the questionnaire while waiting for their clinic consultation. No financial compensation or incentives were offered for participation.

### Statistical analysis

All statistical analyses were conducted using the IBM SPSS Statistics for Windows, version 29.0 (IBM Corp., Armonk, NY, USA). Continuous data were summarised as means and standard deviations given their normal distribution. Categorical data were presented as percentages. Descriptive statistics were used to present sociodemographic profiles, clinical characteristics and SMBG practices.

Univariate analysis (unadjusted odds ratios) was used to determine a single factor’s relative contribution to the performance of SMBG among patients. Factors with P-values of <0.25 or those deemed clinically important were included in the multivariate logistic regression model.

Multivariable logistic regression was used to determine the factors associated with SMBG performance, and the results were reported as adjusted odds ratios (AORs). The independent variables were age, marital status, smoking status, physical activity, duration of diabetes, diabetes treatment, the presence of diabetes complications, history of hospitalisation and diabetes education. A P-value of <0.05 was considered statistically significant.

## Results

A total of 434 eligible patients were recruited for the study, among whom 38 declined to participate, yielding a response rate of 91.2% (396/434). Specifically, 320 participants (80.8%) were recruited from four government health clinics while 76 participants (19.2%) were recruited from a private general practice clinic in Selangor, Putrajaya and Kuala Lumpur.

The mean age of the participants was 53.59±10.88 years (Table 1). The majority were Malay (73.7%), followed by Indian (16.2%) and Chinese (8.3%). Most participants (90.9%) had attained at least secondary-level education. Approximately 58.8% belonged to the B40 national household income category. Conversely, 51.3% of the participants had diabetes for at least 5 years and above. Insulin was used as part of diabetes treatment in 22.2% of the participants. The majority had never attended structured diabetes education sessions (n=278, 70.2%).

**Table 1:** Sociodemographic and clinical characteristics of the participants (N=396).

Variable		n	%	Mean ± SD
**Age, year**				53.59+10.88
Sex	Male	193	48.7	
	Female	203	51.3	
	Malay	292	73.7	
Ethnicity	Chinese	33	8.3	
	Indian	64	16.2	
	Others	7	1.8	
	Married	309	78.0	
Marital status	Single	28	7.1	
	Separated/divorced	25	6.3	
	Widowed	34	8.6	
	No formal education	11	2.8	
Educational level	Primary education	25	6.3	
	Secondary education	205	51.8	
	Tertiary education	155	39.1	
	B40	233	58.8	
Household income	M40	135	34.1	
	T20	28	7.1	
Smoking status	Yes	59	14.9	
	No	337	85.1	
Physical activity	Yes	248	62.6	
	No	148	37.4	
	<1	26	6.6	
Duration of diabetes, year	1-5	167	42.2	
	5-10	131	33.1	
	>10	72	18.2	
	Diet and exercise	38	9.6	
Current diabetes treatment	Tablet only	270	68.2	
	Use of insulin	88	22.2	
Presence of diabetes complications	Yes	143	36.1	
	No	253	63.9	
History of hospitalisation	Yes	42	10.6	
	No	354	89.4	
Diabetes education	Yes	118	29.8	
	No	278	70.2	

This table presents the baseline characteristics of the adults with T2D recruited across the five primary care clinics. Data are presented as frequencies and percentages, except for age, which is presented as means and standard deviations. T2D: type 2 diabetes, standard deviation: SD, B40: bottom 40% income group, M40: middle 40% income group, T20: top 20% income group.

### Prevalence of SMBG performance

Among the participants, 59.3% (n=235) reported currently performing SMBG, while 19.2% (n=76) had performed SMBG previously but were not doing so at the time of the study. Among those performing SMBG, 61.4% recorded their results, and more than half (57.2%) did not make treatment adjustments based on their glucose readings (Table 2).

**Table 2:** Performance of SMBG among the participants (N=396).

Variable		n	%
	Yes, I am doing it.	235	59.3
Do you perform SMBG?	No, I have never done it before.	85	21.5
	No, but I have done it before.	76	19.2
Recording of SMBG readings (n=311)	Yes	191	61.4
	No	120	38.6
Adjusting treatment based on SMBG readings (n=311)	Yes	133	42.8
	No	178	57.2

This table summarises the prevalence of current and past SMBG performance. For the participants performing SMBG or those who had practised it before (n=311), the table further details practices regarding the recording of glucose levels and subsequent treatment adjustments. SMBG: self-monitoring of blood glucose.

### Factors associated with SMBG performance

Table 3 shows the multivariable logistic regression examining the factors associated with SMBG performance. The participants aged 50-59 years (AOR=2.80, 95% CI=1.03-7.62, P=0.045) and 60-69 years (AOR=3.43, 95% CI=1.25-9.38, P=0.017) were more likely to perform SMBG than those aged >70 years. The single (AOR=0.28, 95% CI=0.08-0.99, P=0.049) and separated/ divorced participants (AOR=0.15, 95% CI=0.04-0.56, P=0.005) were less likely to perform SMBG than the widowed participants. Insulin use was significantly associated with SMBG performance (AOR=7.02, 95% CI=2.44-20.19, P<0.001). In contrast, the participants with diabetes complications were significantly less likely to perform SMBG (AOR=0.57, 95% CI=0.33-0.98, P=0.043).

**Table 3:** Multivariable logistic regression analysis of the factors associated with SMBG performance (N=396).

		OR (95% CI)	P-value	AOR (95% CI)^*^	P-value
Age, year	<40	0.91 (0.34-2.42)	0.850	2.17 (0.64-7.31)	0.213
	40-49	0.73 (0.31-1.73)	0.480	1.10 (0.39-3.13)	0.861
	50-59	2.05 (0.87-4.83)	0.100	2.80 (1.03-7.62)	0.045
	60-69	2.92 (1.20-7.10)	0.020	3.43 (1.25-9.38)	0.017
	>70	1.00		1.00	
Sex	Male	1.00		-	
	Female	1.26 (0.84-1.88)	0.260	-	
Ethnicity	Malay	1.00		-	
	Chinese	0.63 (0.30-1.29)	0.210	-	
	Indian and others	1.09 (0.64-1.86)	0.750	-	
Marital status	Married	0.50 (0.22-1.14)	0.098	0.65 (0.26-1.62)	0.352
	Single	0.20 (0.07-0.60)	0.004	0.28 (0.08-0.99)	0.049
	Separated/divorced	0.12 (0.04-0.39)	<0.001	0.15 (0.04-0.56)	0.005
	Widowed	1.00		1.00	
Educational level	No formal education	0.87 (0.25-2.96)	0.820	-	
	Primary education	0.67 (0.29-1.56)	0.350	-	
	Secondary education	1.18 (0.77-1.80)	0.460	-	
	Tertiary education	1.00		-	
Income	B40	1.00		-	
	M40	0.89 (0.58-1.37)	0.590	-	
	T20	0.55 (0.25-1.20)	0.130	-	
Smoking status	Yes	1.00		1.00	
	No	2.25 (1.28-3.94)	0.005	0.62 (0.33-1.18)	0.142
Physical activity	Yes	1.00		1.00	
	No	0.65 (0.43-0.98)	0.040	1.59 (0.97-2.60)	0.065
Duration of diabetes, year	<1	0.26 (0.10-0.67)	0.005	0.81 (0.26-2.52)	0.710
	1-5	0.32 (0.18-0.59)	<0.001	0.93 (0.43-2.01)	0.847
	5-10	0.85 (0.44-1.61)	0.610	1.32 (0.62-2.81)	0.465
	>10	1.00		1.00	
Diabetes treatment	Diet and exercise	1.00		1.00	
	Tablet only	1.08 (0.55-2.12)	0.831	1.51 (0.70-3.28)	0.293
	Use of insulin	6.33 (2.63-15.28)	<0.001	7.02 (2.44-20.19)	<0.001
Presence of diabetes complications	Yes	2.90 (1.85-4.56)	<0.001	0.57 (0.33-0.98)	0.043
	No	1.00		1.00	
History of hospitalisation	Yes	2.38 (0.20-0.88)	0.020	1.09 (0.45-2.61)	0.854
	No	1.00		1.00	
Diabetes education	Yes	2.33 (1.46-3.72)	<0.001	0.71 (0.41-1.25)	0.237
	No	1.00		1.00	

This table displays the association between the sociodemographic and clinical characteristics and performance of SMBG.

* AOR model was adjusted for age, marital status, smoking status, physical activity, duration of diabetes, treatment regimen, diabetes complications, hospitalisation history and diabetes education. OR: unadjusted odds ratio, AOR adjusted odds ratio, CI: confidence interval. P<0.05 was considered statistically significant.

### Barriers and facilitators of SMBG performance

**Figure 1:**
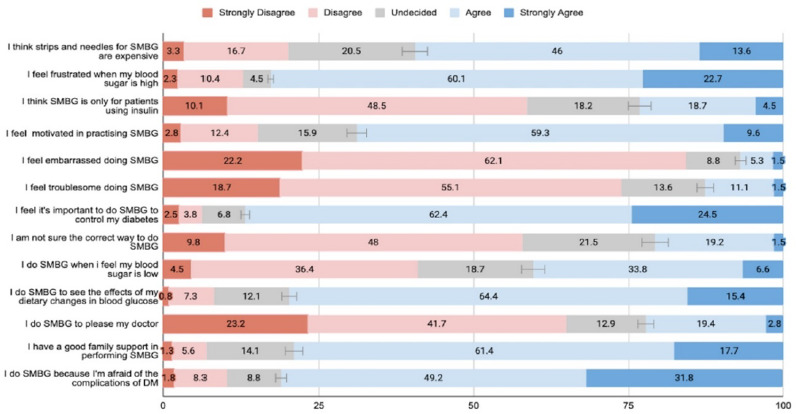
Barriers and facilitators of performing SMBG.

The high cost of test strips and lancets was the main barrier identified, with 59.6% of the participants agreeing that these materials were expensive (Figure 1). Approximately 82.8% reported feeling frustrated when confronted with high blood sugar readings. Nevertheless, 68.9% of the participants reported being motivated to practice SMBG. Most participants (86.9%) perceived SMBG as important in controlling diabetes. The participants performed SMBG due to fear of diabetes complications (81%) and to observe the effects of det on their blood glucose (79.8%). Conversely 79.1% of the participants had good family support in performing SMBG.

## Discussion

Our study found that 59.3% of the participants were currently performing SMBG, and 19.2% had practiced it previously. The middle-aged adults (50–69 years) and insulin users were significantly more likely to perform SMBG, while the participants with diabetes complications were significantly less likely to do so. The main barriers to SMBG included the high cost of strips and needles and emotional frustration with elevated glucose reading, whereas the facilitators included personal motivation, belief in the importance of SMBG, fear of diabetes complications, good family support and the desire to monitor dietary effects on blood glucose.

The prevalence of SMBG in this study (59.3%) is higher than earlier Malaysian reports (15.3%)^11^ but remains lower than rates in Norway (70%)^15^ and Australia (81.7%–88.4%).^16,17^ These higher rates in international cohorts are largely attributed to die widespread availability of structured diabetes education and the implementation of nationwide subsidies for glucometer strips. Although our findings showed an improvement in local SMBG uptake, SMBG is not a routine practice among patients with diabetes in Malaysia.

Despite the relatively high prevalence, the clinical utility of SMBG in our cohort was limited, as more than half of the participants (57.2%) did not adjust their treatment based on glucose readings. This is important, as previous studies have shown that structured SMBG, when combined with self adjustment of treatment, can lead to significant improvements in glycaemic control.^18^ One possible explanation for the low clinical utility is that patients on oral hypoglycaemic agents may be unable to self-modify medications without medical supervision, while patients on insulin may lack the knowledge or confidence to independently adjust insulin doses. This gap is likely compounded by the low prevalence of diabetes education among the patients with diabetes in our study (29.8%), reflecting limited diabetes education coverage in Malaysia. There is a need for wider availability, accessibility and implementation of patient education for patients with diabetes.

Age was an important determinant of SMBG performance. In contrast, Zahari Sham et al. and Mastura et al. reported no significant association between age and SMBG.^7,11^ In our study, the middle-aged adults (50–59 years and 60–69 years) were significantly more likely to monitor their glucose than the older adults aged >70 years. This suggests that SMBG practice may be shaped by life stage. One possible explanation is that middle-aged adults may have higher health literacy and ability to perform SMBG than older adults.^19^ The presence of more structured daily routines and enhanced financial stability during middle age may facilitate the regular incorporation of SMBG into their self-care practices. In contrast, younger adults are less likely to perform SMBG often due to more competing life priorities, less predictable daily schedules, more chaotic lifestyles or lower perceived disease severity.^20^ Older adults may face physical limitations (e.g. impaired vision or neuropathy) or cognitive decline that can hinder SMBG practice.^21^ Our findings differ from an Australian report wherein adults aged <64 years with T2D were found to be less likely to engage in recommended self-care behaviours than older adults aged >64 years.^22^ This discrepancy may be attributed to differences in the population and healthcare context; the Australian study included a larger proportion of individuals with a longer disease duration and potentially greater exposure to structured diabetes education, which may have enhanced SMBG adherence among older adults.^22^

Family support has been shown to play a key role in diabetes self-management. Supportive family behaviours are associated with better treatment adherence among adults with T2D.^23^ Our study found that marital status was associated with SMBG performance. Compared with the widowed individuals (reference group), those who were single or separated/divorced were less likely to perform SMBG, while being married showed a positive but non-significant association. This is consistent with the findings of Dixon Jones,^24^ showing that marital status can influence SMBG practices, particularly among men, which suggests that spousal support may facilitate better adherence. One possible explanation for our findings is that widowed individuals, although lacking spousal support, might be older and receive support from their children or extended family. Although the sample sizes were unequal and small across the marital status categories in our study, the observed differences between these groups may still reflect meaningful variations in the levels of family or spousal support and the impact on SMBG.

Our study revealed a higher SMBG uptake among the insulin-treated patients, consistent with other local and international reports.^11,15,25^ This could be because insulin users may perceive themselves to be at a higher risk of complications and are often encouraged by healthcare providers to monitor glucose levels regularly. Our analysis also showed that the patients with diabetes complications were less likely to perform SMBG, comparable to previous Malaysian findings.^7,11^ One possible explanation is that complications such as neuropathy and retinopathy may pose physical barriers to performing SMBG. Psychological distress arising from longstanding diabetes and poor glycaemic control may reduce motivation, leading to disengagement from self-care practices.^26^

Our study failed to show an association between diabetes education and SMBG performance, contrasting with the current literature. Previous studies have reported that patients who attended diabetes education have better knowledge about SMBG, are more engaged in performing SMBG and have better SMBG compliance and glycaemic control.^21,22^ The absence of association between diabetes education and SMBG performance in our study could be attributed to the small proportion of participants who received diabetes education. Our findings highlight a gap in improving the quality and implementation of structured diabetes education to strengthen patients’ ability to perform and interpret SMBG readings, make informed treatment decisions and ultimately achieve better glycaemic targets.

In our study, cost was the main barrier to performing SMBG because more than half of our participants belonged to the low-income category, where affordability remained a central concern. This contrasts with the findings of a Hong Kong study,^27^ where the sample was drawn from one of the wealthiest districts (Wanchai). This difference suggests that financial constraints are a key obstacle to effective diabetes self-management in the local context. Most participants also expressed frustration over persistently high glucose readings as a major psychological barrier, consistent with the findings by Ong et al.^13^ The patients often interpreted these high readings as a personal failure, which caused substantial discouragement and led them to avoid future testing to escape these negative emotions.

Our participants reported several important facilitators. Most participants agreed that SMBG was important in controlling diabetes, reflecting strong motivation and a positive attitude towards diabetes self-care. Fear of diabetes-related complications further reinforced SMBG practice in our study, consistent with the findings by Hu et al.,^28^ who reported that the perceived susceptibility to and severity of complications significantly influenced self-care behaviours among patients with T2D in China. Participants also described receiving emotional encouragement, reminders and practical assistance from family members, which further supported their adherence. Many participants performed SMBG to observe the impact of dietary changes, suggesting that SMBG is a tool not only for monitoring glycaemic control but also for guiding lifestyle adjustments. These findings support the integration of family-centred education and behavioural interventions into diabetes management programmes.^29^

### Strengths and limitations

A major strength of this study is the inclusion of a broad range of participants recruited from both government and private general practitioner clinics across urban, suburban and rural settings. Our research instruments were adapted from previous studies conducted in Malaysia and are culturally appropriate and relevant to the Malaysian context.

Nevertheless, our study also has several limitations. One limitation is the use of convenience sampling, which may introduce selection bias, as the researchers recruited patients at their convenient time. However, there would not be any substantial difference in the prevalence estimates, as patients came for diabetes follow-up in both morning and afternoon sessions in the clinics. Individuals with higher literacy levels may have been more inclined to complete the self-administered questionnaire, potentially resulting in response bias. While the inclusion of five diverse sites enhances the study’s relevance, the findings may not be fully generalisable to the entire Malaysian population or to patients who seek care exclusively in non-participating private healthcare sectors. Another limitation is that internal consistency (Cronbach’s alpha) was not measured for questionnaire validation. SMBG performance was self-reported and binary, lacking objective verification through glucometer logs or a defined frequency threshold for clinical meaningfulness. Diabetes education was limited to a binary self-reported variable. While this variable was defined as the completion of a formal session with a doctor or certified nurse, the study did not capture specific data regarding the frequency, duration or quality of the education received. Data on test strip ownership, cost subsidies and health literacy were also not collected. These factors may act as residual confounders that can influence the association between demographics and SMBG practice.

### Implications and recommendations

Our findings have important implications for diabetes care in Malaysia. Cost remains the primary barrier for our participants. Implementing government subsidies for test strips and lancets is a critical policy step towards health equity, especially for the B40 income group. While the feasibility of nationwide subsidies depends on fiscal resources, targeted financial aid for high-risk patients or those on insulin could reduce the long-term socioeconomic burden of diabetes complications. Structured diabetes education should be expanded and implemented effectively to equip patients with the knowledge needed to perform and act upon SMBG results, especially patients treated with insulin. Incorporating family support into diabetes care and addressing emotional distress related to glucose monitoring may further enhance adherence. Future longitudinal studies are recommended to assess the psychological and systemic factors influencing SMBG performance as well as intervention studies to evaluate strategies to improve SMBG uptake and effectiveness.

## Conclusion

SMBG uptake among patients with T2D is moderate but suboptimal. Middle-aged adults and insulin users are more likely to perform SMBG, while those with diabetes complications remain less engaged. Financial constraints and emotional frustration are the primary barriers to adherence. Personal motivation, family support and belief in SMBG importance are the main facilitators. Strengthening structured diabetes education and implementing policies to reduce the cost of monitoring supplies are essential to enhance diabetes self-care and improve glycaemic control.

## Data sharing statement

Data are available upon request from the corresponding author.

**How does this paper make a difference in general practice?**

- Our study shows that self-monitoring of blood glucose (SMBG) is suboptimal among patients with diabetes in primary care, highlighting a need for strategies to improve selfmonitoring and self-care.
- Our study highlights priority groups for targeted support in general practice by showing that middle-aged patients and those on insulin are more likely to perform SMBG, while patients with diabetes complications are less engaged in monitoring and may require more proactive interventions.
- Our findings show that cost and emotional frustration with high glucose readings hinder SMBG, underscoring the need for interventions that improve affordability and provide psychological support to patients with diabetes.
